# Circulating Lipocalin-2 level is positively associated with cognitive impairment in patients with metabolic syndrome

**DOI:** 10.1038/s41598-022-08286-x

**Published:** 2022-03-17

**Authors:** Kanokporn Pinyopornpanish, Arintaya Phrommintikul, Chaisiri Angkurawaranon, Sirinart Kumfu, Salita Angkurawaranon, Uten Yarach, Nida Buawangpong, Nipon Chattipakorn, Siriporn C Chattipakorn

**Affiliations:** 1 Department of Family Medicine, Faculty of Medicine, Chiang Mai University, Chiang Mai, 50200 Thailand; 2 Department of Internal Medicine, Faculty of Medicine, Chiang Mai University, Chiang Mai, 50200 Thailand; 3 Neurophysiology Unit, Cardiac Electrophysiology Research and Training Center, Faculty of Medicine, Chiang Mai University, Chiang Mai, 50200 Thailand; 4 Center of Excellence in Cardiac Electrophysiology Research, Chiang Mai University, Chiang Mai, 50200 Thailand; 5 Department of Radiology, Faculty of Medicine, Chiang Mai University, Chiang Mai, 50200 Thailand; 6 Department of Radiologic Technology, Faculty of Associated Medical Sciences, Chiang Mai University, Chiang Mai, 50200 Thailand; 7 Department of Oral Biology and Diagnostic Sciences, Faculty of Dentistry, Chiang Mai University, Chiang Mai, 50200 Thailand

**Keywords:** Neuroscience, Medical research, Neurology

## Abstract

The association between Lipocalin-2 (LCN2) and cognition in patients with metabolic syndrome (MetS) has not been thoroughly investigated. We aimed to evaluate whether serum LCN2 levels are associated with the alteration of cognitive function in patients with MetS**.** The total of 191 non-demented participants with MetS were enrolled onto the study in 2015, and a cohort study was conducted in a subpopulation in 2020. After adjustment for sex, age, waist circumference, creatinine levels, and HbA1C, an association between the higher serum LCN2 levels and the lower Montreal cognitive assessment (MoCA) scores was observed (B = − 0.045; 95%CI − 0.087, − 0.004; *p* 0.030). A total of 30 participants were followed-up in 2020. Serum LCN2 levels were decreased in correlation with age (23.31 ± 12.32 ng/ml in 2015 and 15.98 ± 11.28 ng/ml in 2020, *p* 0.024), while other metabolic parameters were unchanged. Magnetic resonance imaging studies were conducted on a subsample of patients in 2020 (n = 15). Associations between high serum LCN2 levels from 2015 and 2020 and changes in brain volume of hippocampus and prefrontal cortex from 2020 have been observed. These findings suggest a relationship between changes of the level of circulating LCN2, cognitive impairment, and changes in brain volume in patients with MetS. However, further investigation is still needed to explore the direct effect of circulating LCN2 on the cognition of MetS patients.

## Introduction

Metabolic syndrome (MetS) refers to a cluster of several pathological conditions that can increase the incidence of type 2 diabetes, cardiovascular conditions, stroke, and neurodegeneration. The features of MetS include obesity, as indicated by excess body fat around the waist, dyslipidemia, hyperglycemia, and hypertension. In addition, much evidence from in vivo and clinical studies has demonstrated a relationship between MetS and cognitive decline^1,2^. However, the underlying mechanisms of links between MetS and cognitive decline are elusive.

The prodromal stage of cognitive decline in humans is called mild cognitive impairment (MCI). Individuals with MCI develop cognitive abnormalities in normal daily function^3,4^. Approximately twenty percent of the people with MCI progress to dementia a few years later^5–7^. Although physical examination can be used to make a diagnosis of cognitive impairment, the clinical signs of MCI have been mainly identified at later stages of cognitive decline or in the early phase of dementia^8^. Therefore, a tool to distinguish the earliest stage of MCI could be of the greatest benefit to patients with dementia in order to intervene in the development of cognitive impairment in MCI patients or to promptly protect patients from the development of dementia. Several underlying mechanisms of MCI and dementia have been proposed including neuroinflammation, oxidative stress, insulin resistance and microvascular abnormalities in the brain^9–12^.

Lipocalin-2 (LCN2), known as neutrophil gelatinase-associated lipocalin, 24p3, or siderocalin, is a small glycoprotein in the lipocalin family^13^. LCN2 acts as an acute phase protein and it is an inducible factor synthesized and secreted by reactive astrocytes, activated microglia, neurons, choroid plexus and endothelial cells in response to inflammatory or injurious insults to the brain^14^. It has been demonstrated that LCN2 acts as a modulatory factor for diverse cellular functions in the brain, including cell death and survival^15,16^, morphology^16^, migration^16,17^, and amplification of inflammatory responses^18–20^. LCN2 also play roles in the regulation of brain iron^21,22^, the innate immune response, and the regulation of neuroinflammation and neurodegeneration^14^. Apart from its molecular properties, LCN2 has been shown to be responsible for several biobehavioral activities, including pain hypersensitivity, cognitive functions, emotion, depression, and anxiety^14^. It has been proposed that circulating LCN2 could be used a biomarker for many conditions, for example, acute kidney injury^23^, cardiovascular diseases^24^, obesity and metabolic syndrome^25^, and various brain disorders, including dementia^26^.

Clinical studies into LCN2 and its relationship with cognitive impairment have mainly been conducted in AD populations, findings indicating potential predictive properties of the protein^26–29^. A recent study demonstrated that the circulating LCN2 level is involved early in AD pathogenesis at preclinical stages and may be an early blood biomarker of amyloid-beta pathology^30^. However, the knowledge of its role in MCI in the MetS condition is still unclear. Therefore, this study aims to evaluate whether circulating LCN2 level is associated with cognitive status in patients with MetS. Furthermore, a 5-year follow up in a random subpopulation was conducted to assess the predictive property of circulating LCN2 level on mild cognitive impairment by assessing cognitive performance and the anatomical structure of the brain and its volume.

## Results

From the total of 191 patients with MetS in 2015, 107 (56.02%) were female, and the average age was 64.53 (SD 8.45). Mean serum LCN2 levels and MoCA score were 29.70 ng/mL (SD 16.75) and 19.42 (SD 4.72), respectively. Demographic data of the participants is described in Table 1.

**Table 1:** Demographic data of all participants in 2015.

Parameters	N = 191
**Clinical parameters**	
Age (years)	64.53 ± 8.45
Female, n (%)	107 (56.02)
History of disease complications	
-Myocardial infarction	39 (20.42)
-Stroke	5 (2.62)
Current smoker, n (%)	5 (2.62)
Current alcohol drinker, n (%)	33 (17.28)
BMI (kg/m^2^)	27.35 ± 5.36
WC (cm)	94.62 ± 12.93
Systolic BP (mmHg)	138.12 ± 17.86
Diastolic BP (mmHg)	74.38 ± 9.84
MoCA score	19.42 ± 4.72
**Biological parameters**	
Hemoglobin (g/dL)	12.43 ± 1.72
WBC (cell/mm^3^)	7,939.61 ± 2,532.32
Triglycerides (mg/dL)	133.48 ± 69.23
LDL-C (mg/dL)	92.69 ± 35.44
HDL-C (mg/dL)	47.54 ± 14.64
FPG (mg/dL)	130.96 ± 54.31
HbA1C (%)	7.38 ± 1.70
HOMA index	2.77 ± 5.11
Creatinine (mg/dL)	1.34 ± 1.44
Serum LCN2 (ng/mL)	29.70 ± 16.75

Data are presented as mean ± SD.

*BMI* Body mass index, *BP* Blood pressure, *FPG* Fasting plasma glucose, *HbA1c* Glycated hemoglobin A1c, *HDL-C* High density lipoprotein cholesterol, *HOMA index*, Homeostasis Model Assessment index, *LDL-C* Low density lipoprotein cholesterol, *LCN2* lipocalin-2, *MoCA* Montreal cognitive assessment, *WBC* White blood cell count, *WC* Waist circumference.

Table 2 shows the association between serum LCN2 levels and MoCA score in 2015. Serum LCN2 levels were negatively associated with the MoCA scores in crude analysis (B = − 0.043; 95%CI − 0.082, − 0.003; *p* 0.004). After adjustment for sex, age, waist circumference, creatinine levels, and HbA1C, a significant association between the higher serum LCN2 levels and the lower MoCA scores was still observed (B = − 0.045; 95%CI − 0.087, − 0.004; *p* 0.030).

**Table 2:** The association between LCN2 and MoCA score in 2015 (Regression analysis).

	MoCA score	B	95% CI		*P* value
Model 1	Serum LCN− 2	− 0.043	− 0.082	− 0.003	0.037
Model 2	Serum LCN− 2	− 0.045	− 0.087	− 0.004	0.030
	Male gender	0.122	− 1.225	1.469	0.858
	Age	− 0.131	− 0.209	− 0.053	0.001
	Waist circumference	0.078	0.027	0.130	0.003
	Creatinine	0.153	− 0.522	0.827	0.656
	HbA1C	− 0.813	− 1.194	− 0.431	< 0.001

HbA1c, Glycated hemoglobin A1c; LCN2, lipocalin-2; MoCA, Montreal cognitive assessment .

**Figure 1:**
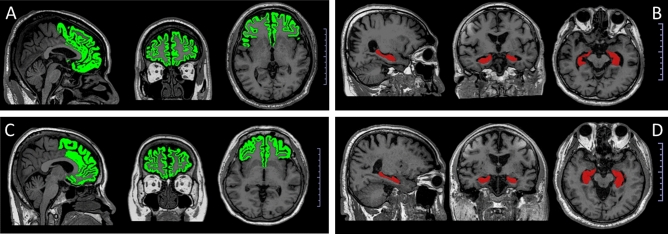
Representation of MRI brain images of participants, the prefrontal cortex identified in green and the hippocampus in red (scale bar, 1 cm × 10 scale); (**A)** the brain of a 59-year-old man with a MoCA score = 25, total prefrontal cortex percentage = 26.9 and circulating lipocalin level in 2020 = 8.59 ng/ml; **(B**) the brain of a 65-year-old man with a MoCA score = 27, total hippocampus percentage = 0.6 and circulating lipocalin level in 2020 = 21.15 ng/ml; (**C)** the brain of a 60-year-old man with a MoCA score = 24, total prefrontal cortex percentage = 23.4 and circulating lipocalin level in 2020 = 21.98 ng/ml; **(D)** the brain of a 60-year-old man with a MoCA score = 25, total hippocampus percentage = 0.49, and circulating lipocalin level in 2020 = 13.07 ng/ml.

Out of the 30 participants in the subpopulation that had been followed up in 2020, fifty percent were female and the mean age in 2015 was 60.1 years (SD 4.63). No significant changes in clinical and biological parameters from 2015 to 2020 was observed, except for serum LCN2 levels which were significantly decreased in 2020, as shown in Table 3. Serum LCN2 levels in 2015 were negatively associated with the volume of the left hippocampus in 2020. In 2020, the levels of LCN2 were significantly related to the lower volume of left and right prefrontal cortices and higher left hippocampal volume. These data are presented in Table 4. Figure 1 shows the representation of the prefrontal cortex and hippocampus from MRI brain of patients with MetS. No association between white matter hyperintensity and LCN2 levels in 2015 and 2020 were observed (*p* 0.536 and 0.095, respectively). There was also no association between specific brain volume and MoCA scores in 2015 and 2020.

**Table 3:** Changes in clinical and biological parameters from 2015 to 2020, comparison using the paired t-test (N = 30).

Parameters	2015 (N = 30)	2020 (N = 30)	*P* value
**Clinical parameters**			
BMI (kg/m^2^)	27.33 ± 4.21	27.23 ± 3.95	0.760
WC (cm)	92.62 ± 11.01	92.68 ± 10.96	0.960
Systolic BP (mmHg)	130.00 ± 14.74	133.83 ± 16.31	0.293
Diastolic BP (mmHg)	74.90 ± 9.24	75.77 ± 10.53	0.708
MoCA score	21.40 ± 3.89	21.53 ± 3.63	0.778
**Biological parameters**			
Hemoglobin (g/dL)	13.13 ± 1.62	13.52 ± 1.99	0.129
WBC (cell/mm^3^)	6,941.03 ± 1,653.99	6,313 ± 2,283.21	0.099
Triglyceride (mg/dL)	131.97 ± 60.48	140.60 ± 88.01	0.409
LDL-C (mg/dL)	87.34 ± 27.30	76.21 ± 25.55	0.056
HDL-C (mg/dL)	49.93 ± 14.63	53.03 ± 15.36	0.086
FPG (mg/dL)	140.27 ± 55.43	131.24 ± 65.49	0.506
HbA1C (%)	7.10 ± 1.29	7.05 ± 1.34	0.817
HOMA IR index	3.08 ± 2.81	9.47 ± 19.04	0.066
Creatinine (mg/dL)	0.94 ± 0.29	1.01 ± 0.29	0.059
Serum LCN2 (ng/mL)	23.31 ± 12.32	15.98 ± 11.28	0.024

Data are presented as mean ± SD.

*BMI* Body mass index, HbA1c, *BP* Blood pressure, *FPG* Fasting plasma glucose, *HbA1c* Glycated hemoglobin A1c, *HDL-C* High density lipoprotein cholesterol, *HOMA index* Homeostasis Model Assessment index, *LDL-C* Low density lipoprotein cholesterol, *LCN2* lipocalin-2, *MoCA* Montreal cognitive assessment, *WBC* White blood cell count, *WC* Waist circumference.

**Table 4:** The association between serum LCN2 levels and brain regions in 2020 (Spearman’s rank correlation).

	Right		Left		Total	
	ρ	*P* value	ρ	*P* value	ρ	*P* value
**LCN2 2015 and lobes**						
Frontal lobe	0.150	0.593	0.150	0.593	0.214	0.442
Parietal lobe	− 0.064	0.819	0.059	0.834	− 0.050	0.859
**LCN2 2015 and specific brain regions**						
Prefrontal cortex	0.464	0.081	0.035	0.899	0.242	0.383
Hippocampus	− 0.443	0.098	− 0.686	0.005	− 0.649	0.009
**LCN2 2020 and lobes**						
Frontal lobe	− 0.521	0.046	− 0.564	0.028	− 0.591	0.020
Parietal lobe	0.528	0.042	0.430	0.109	0.542	0.036
**LCN2 2020 and specific brain regions**						
Prefrontal cortex	− 0.667	0.006	− 0.542	0.036	− 0.582	0.022
Hippocampus	0.258	0.352	0.555	0.032	0.528	0.043

LCN2, lipocalin-2.

## Discussion

Several clinical studies have reported controversial results about LCN2 levels and cognitive status^37^, however, the present study showed a positive association between circulating LCN2 and cognitive decline in non-demented MetS population. We found that circulating LCN2 levels declined over time. Even though there was no association between the change in LCN2 levels and cognitive performance in 2020, the volume of the prefrontal cortex in 2020 recorded using MRI was negatively associated with LCN2 levels at the same timepoint. Interestingly, the volume of the hippocampus in 2020 was negatively associated with serum LCN2 levels in 2015.

It has been shown that MetS can contribute to cognitive impairment^38^, but the role of LCN2 in cognitive decline in patients with MetS was unknown. The positive finding of an association between circulating LCN2 levels and cognitive decline in our study highlights the possible role of LCN2 as a possibly predictor of MCI in patients with MetS. There are reports that LCN2 is associated with metabolic dysfunction and insulin resistance^39,40^. Interestingly the circulating LCN2 levels among patients with MetS was higher than those without MetS^41^. Thus, there is a possibility that LCN2 could be one of factors involved in the MetS condition, and may contribute to cognitive impairment. However, whether LCN2 has a detrimental or beneficial role in metabolic syndrome is still unclear. In the case of cognitive function, an increase in circulating LCN2 levels tended to be harmful as demonstrated with in vitro and in vivo studies as described in a recent review^37^. Therefore, the higher levels of LCN2 in patients with MetS are not likely to be of benefit to the cognitive status of the patients.

Our results are consistent with a previous study that reported that the circulating LCN2 levels in a cognitive impaired population were higher than a control group with normal cognition^37^. In addition, several clinical studies have been conducted in many cognitive impaired populations to explore the relationship between LCN2 and cognitive status. A positive correlation between circulating LCN2 level and cognitive decline was reported in vascular dementia^19^, late-life depression^42^, and MCI populations^29^. We found that the higher circulating LCN2 levels were associated with lower cognitive testing scores in a non-demented MetS population. A previous study also reported an association between plasma LCN2 levels and another global-score cognitive test, the Mini-Mental State Examination score (MMSE)^29^. However, our findings are contradictory to those from another recent study in preclinical AD which reported no association between LCN2 levels and MMSE^30^ adjusted for age and sex.

Interestingly, the circulating LCN2 levels in our study were significantly decreased in 2020 compared to 2015 without any changes in kidney function, and metabolic status. This finding implies that circulating LCN2 level may decrease with age. This result is in contrast with prior cross-sectional studies, which reported that circulating LCN2 concentrations are increased with age^43,44^. Aging is associated with increased levels of many circulating cytokines and proinflammatory markers^45^. For example: elevated levels of interleukin (IL)-1, IL-6, and tumor necrosis factor-α are associated with an older population. However, we found that LCN 2 levels significantly decreased when people got older. Our study may not represent the general aging population as the population in our study involved MetS.

A negative association between circulating LCN2 levels in 2015 and the volume of left, but not right, hippocampus in 2020 might imply the predicting role of LCN2 for brain changes in patients with MetS. Left and right hippocampus are associated with different functions, the left hippocampus being related to verbal memory, and the right hippocampus to spatial memory^46^. These differing functions could explain why the association was found only with one, not two sides of hippocampus. However, a limitation to this study is that we did not have the brain imaging in 2015 to compare therefore we could not prove this predictive property. Nonetheless, in 2020, circulating LCN2 levels were negatively associated with the prefrontal cortex in the same year. This brain region found to be associated with LCN2 levels were congruent with the finding from a prior study in preclinical AD^30^. That study demonstrated a possible link between circulating LCN2 levels and the ability to carry out executive functions which are controlled by the prefrontal cortex. There was a significant positive association between LCN2 in 2020 and the left hippocampus in the same year. This might imply that LCN2 could play different roles in different brain regions and it might act differently over time as the overall levels of LCN2 were lower in 2020. Our findings suggest that LCN2 could have a predictive role as an early biomarker for cognitive impairment and changes in the prefrontal cortex related to executive function and in the left hippocampus related to verbal memory.

A strength of this study is its relatively large sample size compared to prior studies. In addition, the five-year time span of this cohort study provides more information about the direction of changes of LCN2 levels and its possible direction of association with cognitive decline. However, some limitations of the study need to be taken into account. First, as mentioned earlier, we had no MRI records to establish brain volume in 2015 to compare with the present time (2020), so we cannot tell whether the brain volume had changed. Secondly, we did not have levels of inflammatory cytokines in the present study, so the underlying mechanism cannot be explained. Lastly, no association between MoCA score and brain volume was observed in our study which may be due to the limitation of the sample size in 2020, the overall low levels of MoCA score in the population, or the limitation of the tool that is not a domain specific assessment tool. Further studies with a larger sample size or using a more in-depth neuropsychological test for each specific domain of cognition could provide more information.

In conclusion, our findings suggest that circulating LCN2 levels are positively associated with the reduction in the volume of the prefrontal cortex in this MetS population and changes in volume of the left hippocampus. Further investigation is still needed to explore the direct effect of circulating LCN2 on the cognitive function of patients with metabolic syndrome as well as the mechanisms underlying these changes.

## Methods

### Participants

The study protocol was approved by the ethics committee of the Faculty of Medicine, Chiang Mai University (Nos: 253/2020, 154/2020, and 427/2020). Written informed consent from each participant was also gathered in this study. All methods were performed in accordance with the relevant guidelines and regulations.

**Figure 2:**
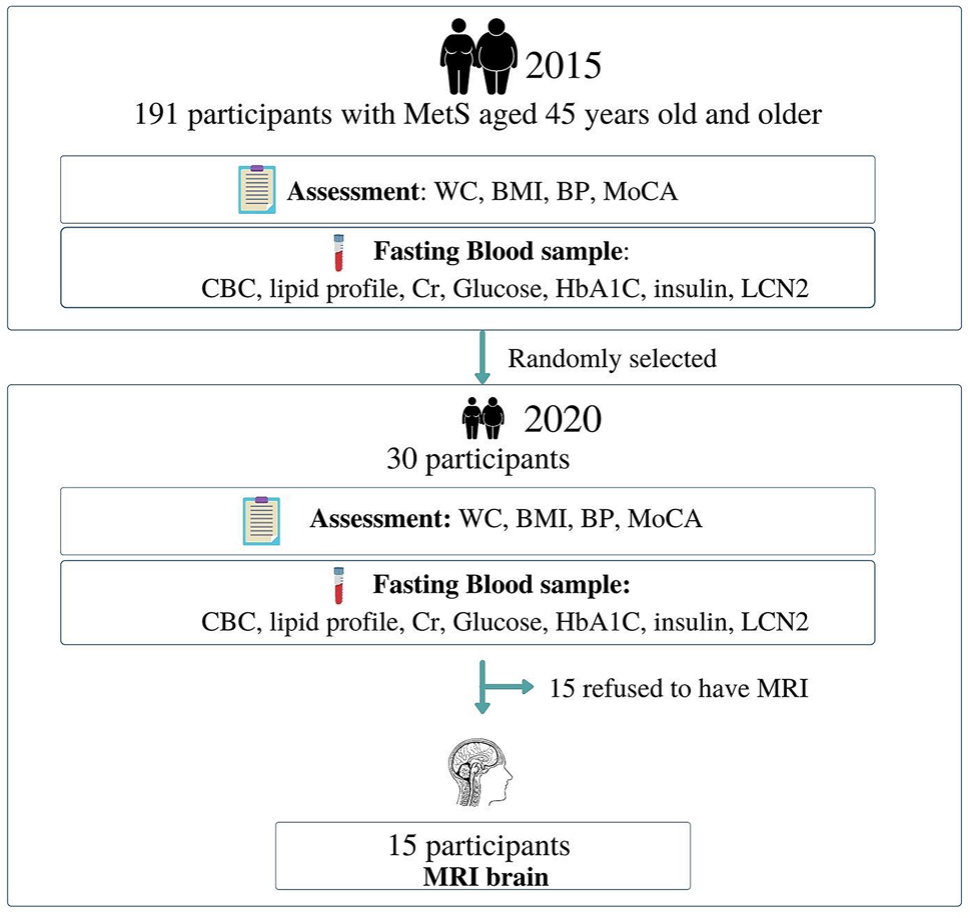
The experimental protocol of the study.

The present study is a sub-study of The Cohort Of patients at a high Risk for Cardiovascular Events (CORE) Thailand registry, which is an ongoing cohort study of patients with a high cardiovascular risk^31^. One hundred and ninety one patients in 2015 were recruited in this study if they met at least three of the following five criteria for MetS (19): (1) elevated waist circumference (WC, ≥ 90 cm in men and ≥ 80 cm in women); (2) elevated triglycerides (TG, ≥ 150 mg/dL) or being treated; (3) reduced high density lipoprotein cholesterol (HDL-C, < 40 mg/dL in men and < 50 mg/dL in women); (4) elevated blood pressure (BP, ≥ 130/85 mmHg) or being treated; (5) elevated fasting plasma glucose (FPG ≥ 100 mg/dL) or being treated. Exclusion criteria of the study included dementia, depression, or had undergone surgery within 3 months. Then, 30 patients with MetS from 2015 were randomly followed up in 2020. The experimental protocol is summarized in Fig. 2.

### Data and specimen collection

All data collection was performed on the same day. These data include history taking, physical examination, and blood collection. History taking was used to collect personal information including age, sex, education, comorbid diseases, history of alcohol drinking and smoking. Data collected from physical examination included WC, blood pressure, body weight and height. Measurements of FPG, HDL-C, LDL-C, TG, insulin, creatinine, and LCN2 levels were performed on fasting venous blood samples collected from each participant. A human LCN2 (RAB0332, Sigma-Aldrich, Darmstadt, Germany) ELISA kit was used to measure serum LCN2 level, in accordance with protocols provided by the test manufacturer.

### Cognitive function assessment

Cognitive function was assessed by using The Montreal Cognitive Assessment (MoCA). The Thai version of the MoCA was used to assess cognitive function as it has high sensitivity and specificity for detecting mild cognitive impairment^32^. The 30 items of the test are to assess multiple cognitive domains including: executive function (adapted trail making test); visuospatial ability (a cube copy task and clock drawing test); language abilities (naming of animals, fluency, and repetition of sentences); abstract thinking (telling similarity between objects); short-term memory (delayed recall); concentration, attention and working memory (digit span task and serial subtraction); and orientation to time and place. The maximum score is 30. The higher scores refer to the better cognitive performance. As the score can be influenced by education, for the Thai version, one point is added to the scores of participants with 6 years of education or less.

### MRI data acquisition and post-processing for hippocampal, prefrontal, and white matter lesion measurements

A magnetic resonance imaging (MRI) study was conducted on a subsample of patients in 2020 (N = 15). All participants underwent a 1.5 T MRI (GE Medical Systems, Waukesha, WI). Imaging protocol consists of the following sequences: a sagittal three-dimensional (3D) IR-SPGR T1-weighted sequence (170 slices, voxel size: 1.00 × 1.00 × 1.00 mm^3^), and an axial 2D T2-FLAIR sequence (27 slices, voxel size: 1.00 × 1.00 × 5.00 mm^3^).

In accordance with the evidence about changes in brain structure from structural imaging of MCI and dementia, apart from volumetric study of each lobe of the brain (frontal lobe, parietal lobe, temporal lobe, occipital lobe, and insula lobe), specific brain regions were chosen for further analysis in this study. These regions included the hippocampus^33^, entorhinal cortex^34^, prefrontal cortex^35^, and precuneus^36^.

All Digital Imaging and Communications in Medicine (DICOM) images from the scanner were converted to the Neuroimaging Informatics Technology Initiative (NIfTI) format using MRIcroGL software (NeuroImaging Tool & Resources Collaboratory, https://www.nitrc.org). T1 IR-SPGR images were used to measure cortical and subcortical structures using Freesurfer6.0 software (https://surfer.nmr.mgh.harvard.edu). For cortical volumes, each individual volume of frontal, parietal, occipital, temporal, and prefrontal regions was normalized by the total cortical volume of cerebrum. The prefrontal volume includes caudalanteriocingulate, lateralorbitofrontal, medialorbitofrontal, parsopercularis, parsorbitalis, parstriangularis, rostralanteriorcingulate, rostralmiddlefrontal, and superiorfrontal regions. For subcortical volume, only the hippocampus was used for investigation, which was normalized against the intracranial cavity. White matter lesion measurement was performed using lesion Brain1.0 software (http://volbrain.upv.es.), in which T1 IR-SPGR and T2-FLAIR images are required.

### Statistical analysis

Statistical analysis was performed using Stata version 16 (StataCorp LLC, College Station, TX, USA). Categorical and Continuous variables were expressed as frequencies and percentages, and mean ± SD, respectively. Multivariable analysis was used to assess the association between LCN2 levels and MoCA score in 2015. Age, gender, WC, and creatinine were all included in the analysis model as potential confounders. Other variables which were significantly associated with MoCA score from univariable analysis were included in the multivariable regression analysis model. The reduced model is shown in Model 2. The associations between each parameter and LCN2 from 2015 and 2020 were determined by using paired t-tests. The association between LCN2 levels and the brain volume in each brain region was determined using Spearman’s rank correlation analysis. A *P* value of less than 0.05 was considered statistically significant.

## Data Availability

The datasets used and/or analyzed in this study are available from the corresponding author on reasonable request.
